# FRUITFULL Is a Repressor of Apical Hook Opening in *Arabidopsis thaliana*

**DOI:** 10.3390/ijms21176438

**Published:** 2020-09-03

**Authors:** Miriam Führer, Angelika Gaidora, Peter Venhuizen, Jedrzej Dobrogojski, Chloé Béziat, Mugurel I Feraru, Jürgen Kleine-Vehn, Maria Kalyna, Elke Barbez

**Affiliations:** 1 Department of Applied Genetics and Cell Biology, University of Natural Resources and Life Sciences Vienna (BOKU), Muthgasse 18, 1190 Vienna, Austria; miriam.fuehrer@students.boku.ac.at (M.F.); angelika_gaidora@yahoo.de (A.G.); peter.venhuizen@boku.ac.at (P.V.); dobrogojski@gmail.com (J.D.); chloe.beziat@ens-lyon.fr (C.B.); mugur.feraru@boku.ac.at (M.I.F.); jkleinevehn@gmail.com (J.K.-V.); mariya.kalyna@boku.ac.at (M.K.); 2 Department of Biochemistry and Biotechnology, Faculty of Agronomy and Bioengineering, Poznań University of Life Sciences, Dojazd 11, 60-632 Poznań, Poland

**Keywords:** apical hook opening, *AGAMOUS-LIKE8*, *FRUITFULL*, IAA, light

## Abstract

Plants adjust their architecture to a constantly changing environment, requiring adaptation of differential growth. Despite their importance, molecular switches, which define growth transitions, are largely unknown. Apical hook development in dark grown *Arabidopsis thaliana* (*A. thaliana*) seedlings serves as a suitable model for differential growth transition in plants. Here, we show that the phytohormone auxin counteracts the light-induced growth transition during apical hook opening. We, subsequently, identified genes which are inversely regulated by light and auxin. We used in silico analysis of the regulatory elements in this set of genes and subsequently used natural variation in gene expression to uncover correlations between underlying transcription factors and the in silico predicted target genes. This approach uncovered that MADS box transcription factor *AGAMOUS-LIKE 8 (AGL8)/FRUITFULL (FUL)* modulates apical hook opening. Our data shows that transient *FUL* expression represses the expression of growth stimulating genes during early phases of apical hook development and therewith guards the transition to growth promotion for apical hook opening. Here, we propose a role for FUL in setting tissue identity, thereby regulating differential growth during apical hook development.

## 1. Introduction

**Figure 1:**
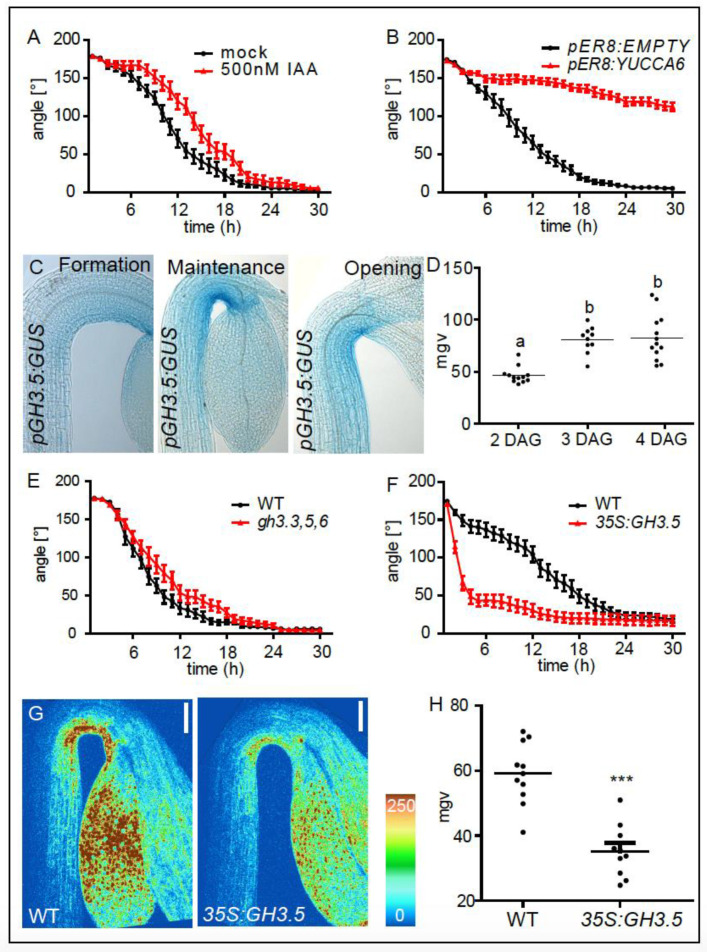
Auxin represses light induced growth transition in apical hooks. (**A**) Kinetics of light triggered apical hook opening in dark grown wild type (WT) seedlings in the maintenance phase (3 DAG) in the presence or absence of 500 nM indole-3-acetic-acid (IAA). (**B**) Kinetics of light triggered apical hook opening in dark grown *pER8:EMPTY* and *pER8:YUCCA6* seedlings in the presence of 10 µM estradiol. (**C**) Expression patterns of *pGH3.5:GUS* in the formation (2 DAG), maintenance (3 DAG), and the opening (4 DAG) phase. (**D**) Graph depicts mean grey values (mgv) of the *pGH3.5:GUS* intensity at the inner side of the hook (statistical significance was tested using a 1-way-ANOVA and a Bonferroni post-hoc test, *p* < 0.05, *n* ≥ 10 apical hooks per developmental stage. Letters indicate different significance classes). (**E**,**F**). Kinetics of light triggered apical hook opening in dark grown WT, *gh3.3,5,6* and *p35S:GH3.5* seedlings in the maintenance phase (3DAG). (**G**) *DR5* promoter activity as shown by *pDR5:GFP* signal intensity in 3-day-old (maintenance phase), dark grown WT and *p35S:GH3.5* seedlings as visualized using confocal microscopy. Color code (blue to red) depicts (low to high) *pDR5:GFP* signal intensity. (**H**) Graph depicts mean grey values of *pDR5:GFP* intensity at the inner side of the hook. Statistical significance was tested using an unpaired Student’s *t*-test, *** *p* < 0.0001, *n* ≥ 10 seedlings per line.

Dicotyledonous plants form an apical hook when seeds are germinating in the soil. It protects the shoot apical meristem and the cotyledons when seedlings are growing through the soil seeking for light [1]. Apical hook development is a very suitable model to study differential growth regulation, due to its non-vital nature during in vitro plant cultivation [1,2,3,4]. During the apical hook formation phase, the PIN- and AUX1/LAX-dependent intercellular auxin transport machinery establishes an auxin signaling maximum at the inner side of the hook [5,6,7,8]. At this site, auxin asymmetrically represses growth, leading to the curvature of the hypocotyl and apical hook formation [5,7,9]. Afterwards, a light sensitive growth promotion machinery induces cell expansion at the inner side of the apical hook enabling its opening (Figure 1A) [2]. Accordingly, the apical hook is a unique model system to address conditional transitions of differential growth repression to promotion [2,10]. Here, we address the molecular pathways that define apical hook opening and aimed to identify transcription factors that steer this differential growth transition.

## 2. Results and Discussion

Besides its role in apical hook formation, asymmetric auxin signaling has also been proposed to maintain the apical hook [11]. However, a direct inhibiting role for auxin in apical hook opening has not been experimentally shown so far. Therefore, we tested whether auxin indeed inhibits the light induced growth transition between the apical hook maintenance and opening phase. Wild type seedlings were grown in the dark until the maintenance phase (3-days-old). We then transferred the seedlings to growth medium supplemented with 500 nM of the natural auxin, indole-3-acetic-acid (IAA) or solvent control (mock) solution (DMSO), exposed them to a light stimulus, and subsequently performed time lapse imaging (Figure 1A,B). Seedlings exposed to exogenous IAA displayed a slower light-induced apical hook opening compared to mock treated seedlings (Figure 1A). Next, we endogenously induced auxin biosynthesis during apical hook opening, using the transgenic estradiol inducible *pER8:YUCCA6* line. Three-day-old dark grown seedlings (apical hooks in maintenance phase) were transferred on estradiol and the light-induced apical hook opening kinetics were monitored. While the empty vector control seedlings (*pER8:EMPTY*) displayed normal opening kinetics, *YUCCA6* induction largely prohibited the light induced apical hook opening, suggesting that the increased auxin biosynthesis prohibits apical hook opening (Figure 1B). This set of data confirms that auxin signaling indeed represses light triggered apical hook opening.

Since auxin and light play a profound role in apical hook opening, it is to be expected that molecular factors that are inversely regulated by both auxin and light may play a crucial role in this growth transition. Previous work described an auxin and light dependent role for GH3.5 in hypocotyl growth [12]. We therefore hypothesized that GH3.5 may also steer the growth transition towards apical hook opening. *GH3.5* encodes an IAA-amido synthase and is known to conjugate the natural auxin IAA to amino acids, thereby decreasing the active pool of free IAA in the cell [13,14]. In agreement, GUS staining of dark grown transgenic *pGH3.5:GUS* seedlings revealed *GH3.5* expression during apical hook development (Figure 1C,D). Apart from the asymmetric *GH3.5* expression, we observed increased expression towards the maintenance and opening phase, suggesting a role in growth transition (Figure 1C,D). The asymmetry and developmentally defined (stage dependent) expression of *GH3.5* is remarkable, because auxin conjugation is rather known to contribute to cellular auxin homeostasis [15]. We tested whether GH3.5, besides its homeostatic role in safeguarding cellular auxin levels, could play an additional role in the transition of differential growth during apical hook opening. We performed time-lapse imaging of dark grown apical hooks in the maintenance phase that were exposed to a light stimulus (Figure 1E,F). *GH3* genes are highly redundant [14] and only *gh3.3,5,6* triple loss-of-function mutants displayed slower apical hook opening, when compared to wild type (Figure 1E). On the contrary, *GH3.5* overexpressing seedlings showed faster apical hook opening compared to WT seedlings (Figure 1F). Since GH3.5 activity is known to adenylate IAA to amino acids and reduces free IAA levels in the cell [13], we assessed the impact of GH3.5 activity on nuclear auxin signaling during apical hook development by crossing a *p35S:GH3.5* line with the auxin signaling output reporter *pDR5:GFP*. Analysis of apical hooks in the maintenance phase (3-day-old dark grown seedlings), revealed that *GH3.5* overexpression substantially decreases *pDR5:GFP* signaling, confirming a negative impact of *GH3.5* on nuclear auxin signaling in the apical hook (Figure 1G,H). These data suggest that GH3.5 stimulates apical hook opening, presumably by decreasing nuclear auxin signaling at the inner side of the hook structure.

In the absence of a light stimulus, apical hooks still open eventually at a slower pace due to a steady decrease in auxin accumulation at the inner side of the hook [2,7]. In accordance, we also observed a faster apical hook opening in dark grown GH3.5 overexpressing seedlings compared to WT seedlings in the absence of light (Figure S1).

**Figure 2:**
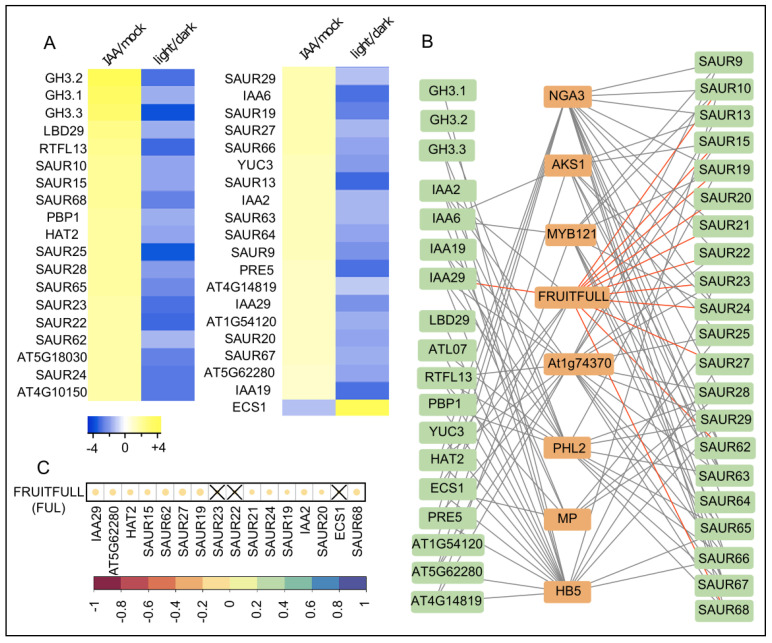
Putative molecular components in differential growth control in apical hook hooks. (**A**) Heat map represents genes that are oppositely regulated by the auxin IAA and light in dark grown seedlings. Cut off values were 2-fold expression difference (−1 ≥ log_2_ ≥ 1) and *p* < 0.001. Color code blue to yellow depicts the log_2_ values of strongly decreased to strongly increased expression compared to the control condition. (**B**) Gene regulatory network depicts the transcription factors (orange boxes) which putatively regulate the auxin- and light-regulated candidate genes (green boxes) according to SeqEnrich [16]. Gray lines connect the transcription factors with their putative target genes. The connecting lines with FUL are highlighted in red. (**C**) Diagram represents the correlation between the expression of FRUITFULL and its target genes in 727 *Arabidopsis thaliana* natural variations [17] Color code (red to blue) depicts highly negative to highly positive Pearson correlation coefficients. The size of the circles also represents the correlation strength between the expression of FUL and its putative target genes. Non-significant (*p* ≥ 0.05) correlations are indicated with a cross.

This set of data confirms the negative role of auxin in light induced apical hook opening. Moreover, this approach shows that auxin and light dependency of molecular factors could hint at a potential role in growth transition during apical hook development. Accordingly, we aimed to identify novel molecular factors for growth transition in apical hooks, depicting genes that are oppositely regulated by auxin and light. Therefore, dark grown WT *A. thaliana* seedlings were transferred (three days after germination (3 DAG) under green light (mimicking dark growth conditions) to growth medium supplemented with DMSO (solvent control) or with 500 nM IAA for 1 h. We then dissected the shoots for subsequent RNA extraction and RNA sequencing (RNA-seq). Analysis of RNA-seq transcriptomics data revealed 241 genes that were significantly (*p* < 0.001) and at least two-fold up- or down-regulated upon auxin treatment (Table S1). We used the genevestigator software to compare our dataset with a previously reported RNA-seq dataset (AT-00706) in which shoot tissue was dissected from 4-day-old dark grown seedlings that were conditionally exposed to light for four hours *(www.genevestigator.com)*. We identified in total 39 genes, displaying opposite regulation by auxin and light (Figure 2A; Table S2). Notably, *GH3.5* is also significantly upregulated by IAA (log_2_FC = 0.94) and downregulated by light (log_2_FC = 1.76). However, *GH3.5* has not been included in the candidate gene list, because of the commonly used threshold cut off (log_2_FC > 1).

We hypothesize that some of these differentially controlled genes may be relevant for growth control and hence could be controlled by transcription factors that define differential growth. In order to identify molecular factors that may define auxin and light dependent growth transitions, we screened in silico for common transcriptional regulators of these auxin- and light-dependent genes, using the previously established SeqEnrich script [16]. Thereby, we identified eight transcription factors, which presumably bind to regulatory elements in upstream regions of the identified genes (statistically significant level of *p* < 0.05) (Figure 2B; Table S3).

Subsequently, we assessed which transcription factors are likely to affect the predicted target genes, using natural variation of gene expression [17]. We assumed that, in case of a simple transcription factor-target gene scenario, the expression of a transcription factor correlates (whether positively or negatively) with the expression of its individual target genes in natural accessions of *A. thaliana*. We therefore calculated the Pearson correlation coefficient for the expression of the eight identified transcription factors and their individual target gene expression in 727 *A thaliana* accessions (Figure 2C; Figure S2). Three out of eight transcription factors, such as AKS1, FRUITFULL and HB5, correlated significantly (*p* < 0.05) with the expression of more than half of their predicted target genes, suggesting a potential role in differential growth control. In agreement, HB5 binds the promoters of the auxin responsive BODENLOS (BDL/IAA12) and EXPANSIN3 and thereby regulates their expression [18,19]. Moreover, HB5 and its target gene EXPANSIN3 are important for cell elongation in hypocotyls [19]. Accordingly, we conclude that our approach is suitable to identify molecular players affecting growth processes.

Interestingly, we also identified AGAMOUS LIKE8(AGL8)/FRUITFULL(FUL) as a transcription factor with a putative role in differential growth. AGL8/FUL expression significantly negatively correlated with a high proportion of its putative target genes in 727 *Arabidopsis thaliana* accessions (Figure 2C; Figure S2). AGL8/FUL is a member of the MADS box transcription factor family which are widely conserved throughout the domain of eukaryotes. Compared to animals, MADS box genes highly expanded during plant evolution. The subfamily of *AGL*s, which contains 42 members in *Arabidopsis*, has been shown to primarily define flower as well as fruit development and, hence, are considered as potential targets for crop improvements [20,21,22,23]. AGL8/FUL has also been reported to be important for the regulation of meristem activity during fruit development, but also for light dependent shoot branching [24,25,26]. However, a potential role of AGL8/FUL in differential growth processes, such as during apical hook development, remains to be assessed. This triggered our interest to further investigate the role of AGL8/FUL in light triggered apical hook opening.

**Figure 3:**
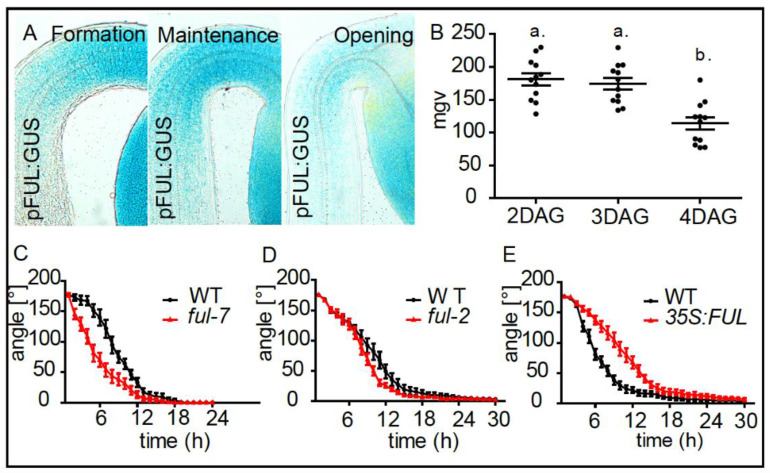
FRUITFULL defines apical hook opening. (**A**) Expression patterns of *pFUL:GUS* in the formation (2 DAG), maintenance (3 DAG) and the opening (4 DAG) phase. (**B**) Graph depicts mean grey values (mgv) of the *pFUL:GUS* intensity at the inner side of the hook (statistical significance was tested using a 1-way-ANOVA and a Bonferroni post-hoc test, *p* < 0.05, *n* ≥ 10 apical hooks per developmental stage. Letters indicate different significance classes). (**C**–**E**) Kinetics of light triggered apical hook opening in dark grown WT, *ful-7*, *ful-2,* and *p35S:FUL* seedlings.

Using the *pFUL:GUS* transcriptional reporter lines, we observed that *AGL8* is expressed during apical hook formation with a decrease towards the opening phase (Figure 3A,B). Time-lapse analysis of light triggered apical hook opening revealed that knock-out mutants *ful-2* and *ful-7* loss-of-function mutants displayed an accelerated apical hook opening (Figure 3C,D), while *AGL8/FUL* overexpressing seedlings displayed a severe delay in apical hook opening when compared to WT seedlings (Figure 3E). This set of data suggests that AGL8/FUL represses growth promoting genes and thereby plays a regulatory role in defining growth transition during apical hook development.

**Figure 4:**
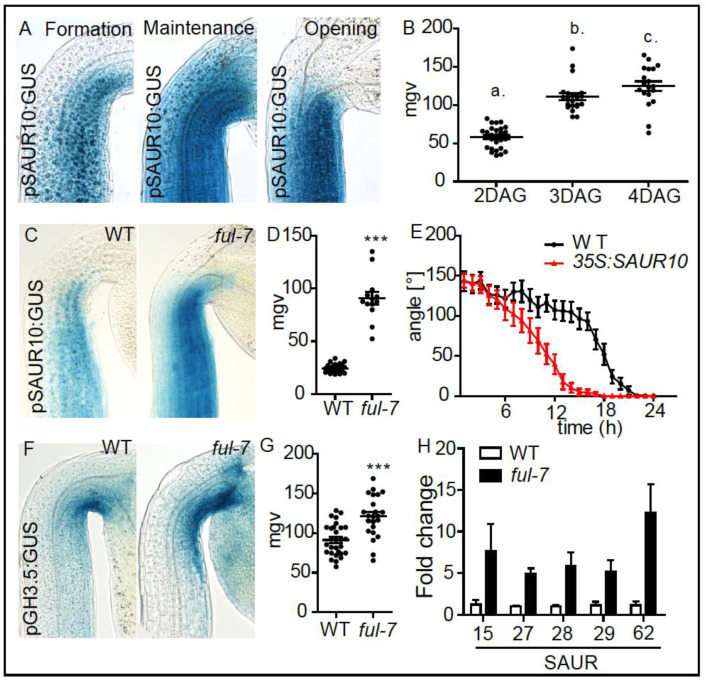
FUL-dependent control of *SAUR10* in apical hooks. (**A**) Expression patterns of *pSAUR10:GUS* in the formation (2 DAG), maintenance (3 DAG) and the opening (4 DAG) phase. (**B**) Graph depicts mean grey values of the *pSAUR10:GUS* intensity at the inner side of the hook (statistical significance was tested using a 1-way-ANOVA and a Bonferroni post-hoc test, *p* < 0.05, *n* ≥ 10 apical hooks per developmental stage. Letters indicate different significance classes). (**C**) pSAUR10 promoter activity in 3-day-old (maintenance phase), dark grown WT and *ful-7* seedlings. (**D**) Graph depicts mean grey values of *pSAUR10:GUS* intensity in the apical hook area. Statistical significance was tested using an unpaired Student’s *t*-test, *** *p* < 0.0001, *n* ≥ 10 seedlings per line. (**E**) Kinetics of light-triggered apical hook opening in dark grown WT and *p35S:SAUR10* seedlings in the early maintenance phase (2-day-old). (**F**) *pGH3.5* promoter activity in 3-day-old (maintenance phase), dark grown WT and *ful-7* seedlings. (**G**) Graph depicts mean grey values of *pGH3.5:GUS* intensity in the apical hook area. Statistical significance was tested using an unpaired Student’s t-test, *** *p* < 0.0001, *n* ≥ 10 seedlings per line. (**H**) Graph depicts Q-PCR measured relative expression levels of putative FUL target genes in dark grown shoots in the maintenance phase (3 DAG) of *ful-7* compared to WT seedlings. The statistical significant difference between WT and *ful-7* was demonstrated using a 2-way-ANOVA test including a Bonferroni post-hoc test. *p* < 0.0001.

It is unknown whether all 16 predicted target genes are indeed direct targets of *AGL8/FUL*. However, based on ChIPseq, AGL8/FUL presumably binds to the promoters of *GH3.5* and *SAUR10* [25]. Moreover, AGL8/FUL was shown to directly bind and consequently represses *SAUR10* [25], which was among our putative target genes. Similar to *GH3.5*, the transcriptional *SAUR10* reporter displayed enhanced expression during maintenance and opening phases of apical hook development (Figure 4A,B). Accordingly, we used these *GH3.5* and *SAUR10* marker lines to further assess the potential role of *AGL8/FUL* during growth transitions in apical hooks.

In order to test whether AGL8/FUL affects *SAUR10* and/or *GH3.5* expression during apical hook development, we investigated *pSAUR10:GUS* and *pGH3.5:GUS* reporter lines crossed into a *ful-7* knock-out mutant. During the maintenance phase, we detected an increased *SAUR10* and *GH3.5* expression in the apical hook of *ful-7* mutants when compared to wild type control lines (Figure 4C,D,F,G). Using qPCR, we also detected higher expression of other *SAUR* genes in dissected apical hook tissues, which were among our list of 16 potential target genes, in the *agl8* mutant background (Figure 4H). This data suggests that AGL8/FUL negatively affects the expression of its target genes, such as *SAUR10,* in apical hooks (Figure 4C,D). Ectopic expression of *SAUR10* caused a faster light triggered apical hook opening compared to wild type seedlings (Figure 4E), which is reminiscent of the overexpression of *GH3.5* (Figure 1F). Our results suggest that de-repression of FUL target genes, such as *SAUR10*, induce faster growth transition during apical hook development.

In this work, we experimentally confirmed the negative impact of exogenous as well as endogenous auxin on apical hook opening which counteracts the effect of light. We used these features to unveil differentially controlled genes. Using in silico predictions of overlapping regulatory elements, we identified novel transcription factors with a presumable role in defining differential growth transitions. We subsequently used natural variation in gene expression as a means to further assess if the expression of the identified transcription factors may positively or negatively correlate with the predicted target genes. These approaches led to the identification of AGL8/FUL as a negative regulator of apical hook opening by repressing growth promoting genes, such as *SAUR10*.

We propose that the decrease in *FUL/AGL8* expression towards the opening phase allows the de-repression of growth promoting genes, such as *GH3.5* and *SAUR10*, which will initiate growth transition and subsequently apical hook opening. Intriguingly, *GH3.5* and *SAUR10* get up- and down-regulated upon IAA and light treatment in dissected hypocotyls, respectively. On the other hand, auxin signaling decreases during light-induced apical hook opening, but *GH3.5* and *SAUR10* both increase during this condition. This observation pinpoints at tissue specific regulation, which warrants further investigation. In this regards, it remains to be seen how precisely FUL/AGL8 integrates developmental and environmental stimuli in order to accurately execute its function during apical hook development.

Interestingly, another MADS box family member, AGL9, also has been described to bind the *GH3.5* promoter [27] and AGL15 interferes with auxin signaling by repressing auxin signaling genes, such as *TIR1, ARF3, ARF6,* and *ARF8* [28,29]. Moreover, light perception impacts on transcription and alternative splicing of various *AGLs* [30]. Accordingly, AGLs could function possibly in part redundantly as a signaling nexus for context specific growth responses by integrating auxin and light signals. AGLs have been studied extensively before, with a strong focus on their role in flower and fruit development. Our findings propose a regulatory role for AGAMOUS LIKE genes in controlling context specific growth transitions.

## 3. Material and Methods

### 3.1. Plant Material

*A. thaliana ecotype* Columbia-0 was used as the wild type. *p35S:GH3.5* (*WES1D*) [12], *gh3.3,5,6* [31] *pGH3.5:GUS* (*pWES1:GUS*) [12] *pER8:EMPTY*, *pER8:YUC6* [32], *ful-2* [33], *ful-7* [25], *p35S:FUL* [25], *p35S:SAUR10* [25], *pSAUR10:GUS* [25], *pFUL:GUS* [25], and *pSAUR10:GUS/ful-7* [25] have been described previously. We crossed the *pDR5:GFP* reporter line with the *35S:GH3.5* line in order to obtain the *pDR5:GFP/p35S:GH3.5* line. We crossed the *pGH3.5:GUS* and *ful-7* line in order to obtain the *pGH3.5:GUS/ful-7* line.

### 3.2. Growth Conditions

Seeds were sterilized with 70% ethanol followed by drying in a sterile hood. Plants were grown under long-day (16 h light/8 h dark) conditions at 20 °C. Seedlings were grown on ½ Murashige and Skoog (MS) in vitro plates supplemented with 0.8% agar. For the depicted treatments, the MS medium was supplemented with 500 nM indole-3-acetic acid (IAA) or 10 µM estradiol. As a mock treatment, an equivalent volume of DMSO was added to the medium. The seeds were stratified at 4 °C for two days in the dark and then were exposed to 8 h of light (22 °C) and subsequently grown vertically in the dark (20 °C) until the corresponding time points. For light-induced apical hook-opening experiments, three days old dark-grown seedlings (apical hooks in maintenance phase) were exposed to constant low light (4 µmol m^–2^ s^–1^) for the duration of time indicated in figure legends. Notably, dark grown *35:SAUR10* seedlings display a premature apical hook opening in the dark. We therefore assessed the apical hook opening in *35:SAUR10* as well as their Col-0-WT controls in the early maintenance phase (2-days-old), when apical hooks were still maintained.

### 3.3. Expression Analysis via GUS Staining

Dark-grown seedlings were collected during the formation, maintenance and/or opening phases of apical hook development as indicated in the text. GUS staining was performed and quantified as described previously [34]. Before staining, seedlings were fixed in 80% cold acetone for 20 min on ice, and subsequently washed three times using MonoQ water before GUS staining. The seedlings were stained for 3 h (*pGH3.5:GUS and pSAUR10:GUS*) and 2 h (*pFUL:GUS*). The stained seedlings were mounted in chloralhydrate, incubated overnight at 4 °C and subsequently imaged using a ZEISS upright light microscope equipped with a camera. Images have been analyzed as described previously [34].

### 3.4. Time-Lapse Imaging of Apical Hook Kinetics

Dark-grown seedlings in the maintenance phase were transferred on in vitro growth plates supplemented with the indicated chemicals and subsequently imaged in a customized light-sealed imaging box equipped with a white light source and a Canon mirror reflex camera (EOS035 Canon Rebel T3i) operated by the Canon EOS utility software. Images were taken and analyzed every hour for a duration of 30 h. Angles between the cotyledons and the hypocotyl axis were measured using the Fiji software (http://rsb.info.nih.gov/ij/). The complementary angle is given in the graphs. At least three independent experiments were performed and the sample size is indicated in the figure legends. To display potential conditional variability among the experimental replicates, all raw measurements have been included in Table S4.

### 3.5. Confocal Microscopy

Confocal microscopy was done with a Leica SP5 or Leica SP2 upright confocal scanning microscope (Leica). Fluorescence signals for GFP (excitation 488 nm, emission peak 509 nm) were detected with a 40× (dry) objective. Fluorescence signal intensity was analyzed using the Fiji software (http://rsb.info.nih.gov/ij/) software and data were statistically evaluated with Microsoft Excel 2010 and Graphpad Prism 5.

### 3.6. RNA Sequencing

Seedlings were grown on sterile nylon meshes for 3 days in the dark until the apical hook maintenance phase. The seedlings on the meshes were then transferred under green light to in vitro growth plates supplemented with 500 nM IAA. After 1 h incubation in the dark, 15–20 shoots per sample were dissected and transferred to a 2 mL Eppendorf tube with 2 glass beads for flash freezing in liquid nitrogen. RNA has been isolated using the innuPREP Plant RNA kit (Analytic Jena, Jena, Germany). Libraries were generated with the NEBNext Ultra II RNA Library Prep Kit for Illumina with poly (A) enrichment, after which the RNA was degraded using Ribozero (New England Biolabs, Ipswich, MA, USA). The sequencing was performed on an Illumina HiSeq2500 with 250 bp paired ended fragments.

A total of nine single-end 50 bp long read data-sets, three biological replicates per sample, were obtained from a single lane of Illumina sequencing. Reads were mapped to the *Arabidopsis thaliana* reference genome TAIR10 (The *Arabidopsis* Genome Initiative 2000) using TopHat v2.0.13 [35] allowing for a maximum of two mismatches. Minimum and maximum intron lengths and minimum anchor length were fixed at 60, 6000, and 12 nt, respectively, and the --b2-very-sensitive and --microexon-search options were enabled. Pearson correlation coefficients between the replicates were calculated using the transcript expression values obtained by StringTie v1.0.4 [36]. Transcript quantification was limited to the TAIR10 annotated transcripts, excluding the chloroplast and mitochondrial chromosomes. Good correlation was obtained for the replicates of all three samples (*R*^2^ > 0.95 or higher).

Differentially expressed genes (DEG) were identified using Cuffdiff 2 [37], with default parameters, for the following comparison: WT + mock vs. WT + IAA (Table 1).

The raw sequencing data has been deposited at the Sequence Read Archive (SRA) of NCBI (SAMN14353622 and SAMN14353623).

### 3.7. qRT-PCR

We dissected shoots from dark grown seedlings in the maintenance phase (3DAG) and transferred them to a 2 mL Eppendorf tube with 2 glass beads for flash freezing in liquid nitrogen. For each sample, 15–20 shoots were pooled. For each line, 3 biological replicates were grown, extracted and analyzed. The tissue was ground using a milling machine (Retsch) after which the RNA was extracted using the InnuPREP Plant RNA Kit (Analytic Jena, Jena, Germany). We synthesized cDNA using the iSCRIPT cDNA Synthesis Kit from Bio-Rad and according to the manufacturer’s recommendation. Q-PCR was carried out in a C1000 Touch Thermal Cycler equipped with the CFX96 Touch Real-Time PCR Detection System (Bio-Rad, Hercules, CA, USA), using a Takyon qPCR Kit for SYBER assay (Eurogentec, Liège, Belgium) and according to the manufacturer’s recommendation. We normalized the expression of the target genes with the expression of the Eif4a and UBL5 household genes.

### 3.8. In Silico Analysis

For the performance of the in silico analysis mentioned in the manuscript, we made use of the Genevestigator (Experiment AT-00706 [38]) (https://genevestigator.com), Cytoscape (https://cytoscape.org) and the Seqenrichtools as described before [16,39,40].

### 3.9. Data Analysis

We used Excel to organize data and MVapp (https://mvapp.kaust.edu.sa) [41] and the GraphPad Prism 5 software for statistical analysis and graphing. For statistical analysis of the raw data, we used Student’s *t*-tests or one/two-way ANOVAs and Bonferroni post-hoc tests as indicated in the figure legends. The most representative images are shown throughout the article. The experiments have been performed in triplicates or more.

## Figures and Tables

**Table 1:** Differentially expressed genes (DEG) between mock treated and IAA treated shoots.

	DEG	Up-Regulated	Down-Regulated
WT + mock vs. WT + IAA	241	159	82

## Supplementary Materials

The following are available online at https://www.mdpi.com/1422-0067/21/17/6438/s1. Figure S1: Kinetics of apical hook development in Col-0-WT and *p35S:GH3.5* dark grown seedlings in the absence of a light stimulus. Figure S2: Correlation between the expression of the transcription factors and their putative target genes in 727 *Arabidopsis thaliana* natural variations. Table S1: Differential gene expression of auxin and mock-treated dark grown hypocotyls. Table S2: Genes displaying inverse regulation by light and auxin as well as the enrichment of regulatory elements in candidate gene list. Table S3: Expression of the transcription factor candidates and their putative target genes in 727 *A. thaliana* natural variations. Table S4: Raw data measurements of the individual apical hook kinetics replicates.

## Abbreviations

AGL	AGAMOUS-LIKE
FUL	FRUITFULL
IAA	indole-3-acetic-acid
DAG	days after germination
mgv	mean gray value
FC	fold change
DEG	differentially expressed genes
3 DAG	3 days after germination
