# Supplementary material for: FRUITFULL Is a Repressor of Apical Hook Opening in Arabidopsis thaliana
Source: Int J Mol Sci. 2020 Sep 3;21(17):6438. doi: 10.3390/ijms21176438 (PMC7504503; doi:10.3390/ijms21176438)
Supplement: Supplementary file 1 [file ijms-21-06438-s001.zip › supplemental files/Sup Figure_2.pdf]

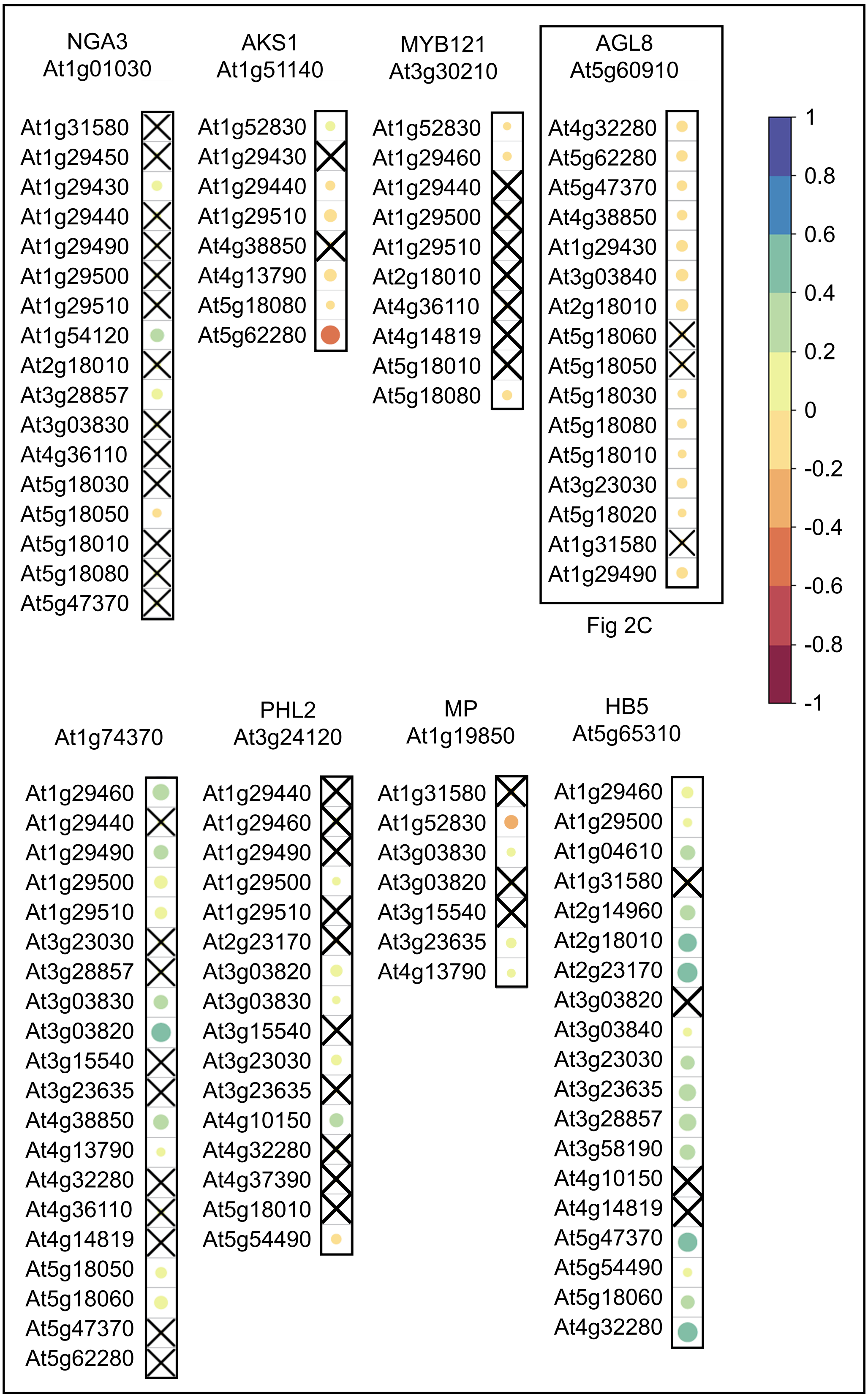

Supplemental Figure 2

Diagrams represents the correlation between the expression of the transcription factors identified by SeqEnrich (Becker et al., 2017) and their target genes in 727 *Arabidopsis thaliana* natural variations (Kawakatsu et al., 2017). Colour code (red to blue) depicts highly negative to highly positive Pearson correlation coefficients. Non-significant ($p > 0.05$) correlations are indicated with a cross.
