# Supplementary material for: FRUITFULL Is a Repressor of Apical Hook Opening in Arabidopsis thaliana
Source: Int J Mol Sci. 2020 Sep 3;21(17):6438. doi: 10.3390/ijms21176438 (PMC7504503; doi:10.3390/ijms21176438)
Supplement: Supplementary file 1 [file ijms-21-06438-s001.zip › supplemental files/Sup Figure_1.pdf]

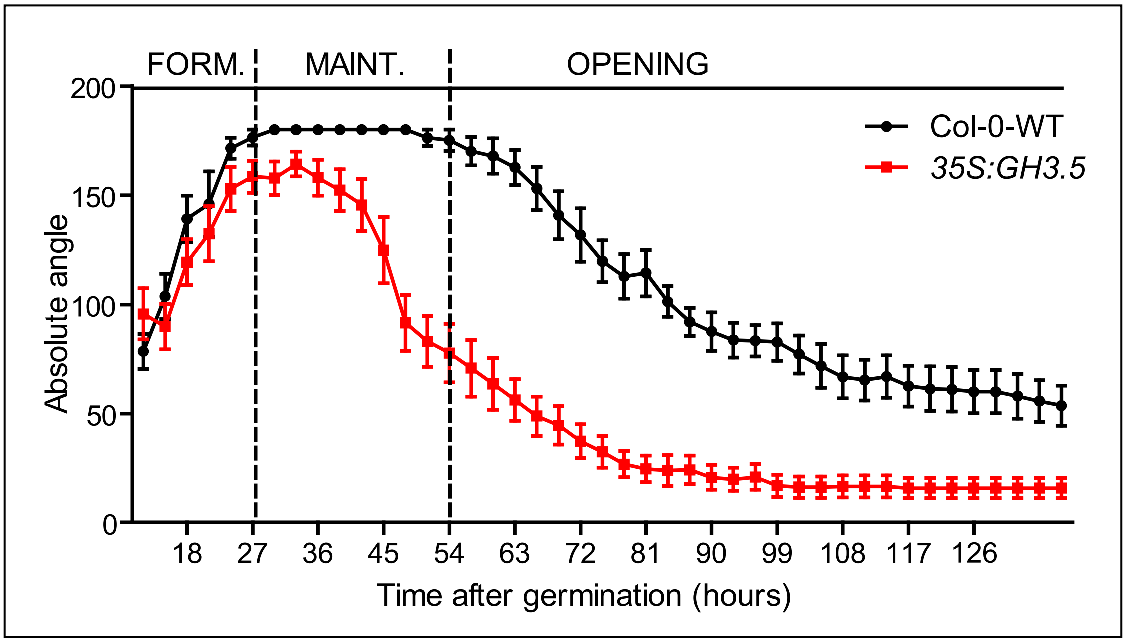

Supplemental Figure 1
Kinetics of apical hook development in Col-0-WT and *35S:GH3.5* dark grown seedlings from germination onwards.
Abbreviations: FORM: formation phase; MAINT: maintenance phase; OPENING: opening phase.
